# The Secret Life of the Inhibitor of Virus Replication

**DOI:** 10.3390/v14122782

**Published:** 2022-12-14

**Authors:** Peter Palukaitis, Masoud Akbarimotlagh, Eseul Baek, Ju-Yeon Yoon

**Affiliations:** 1 Department of Horticulture Sciences, Seoul Women’s University, Seoul 01797, Republic of Korea; 2 Plant Pathology Department, Faculty of Agriculture, Tarbiat Modares University, Tehran 14115-111, Iran; 3 Department of Plant Protection and Quarantine, Jeonbuk National University, Jeonju 54896, Republic of Korea; 4 Department of Agricultural Convergence Technology, Jeonbuk National University, Jeonju 54896, Republic of Korea

**Keywords:** defense response factors, solanaceous, tobacco, Arabidopsis, inhibitor of virus replication, cyclosome, anaphase-promoting complex 7, protein structures

## Abstract

The inhibitor of virus replication (IVR) is an inducible protein that is not virus-target-specific and can be induced by several viruses. The GenBank was interrogated for sequences closely related to the tobacco IVR. Various RNA fragments from tobacco, tomato, and potato and their genomic DNA contained IVR-like sequences. However, IVRs were part of larger proteins encoded by these genomic DNA sequences, which were identified in Arabidopsis as being related to the cyclosome protein designated anaphase-promoting complex 7 (APC7). Sequence analysis of the putative APC7s of nine plant species showed proteins of 558-561 amino acids highly conserved in sequence containing at least six protein-binding elements of 34 amino acids called tetratricopeptide repeats (TPRs), which form helix–turn–helix structures. The structures of Arabidopsis APC7 and the tobacco IVR proteins were modeled using the AlphaFold program and superimposed, showing that IVR had the same structure as the C-terminal 34% of APC7, indicating that IVR was a product of the *APC7* gene. Based on the presence of various transcription factor binding sites in the APC7 sequences upstream of the IVR coding sequences, we propose that IVR could be expressed by these *APC7* gene sequences involving the transcription factor SHE1.

## 1. Introduction

The recruiting or pirating of host proteins to support the replication or movement of plant viruses is a well-characterized form of parasitism, as mutant recessive genes encoding many of these factors fail to function in enabling virus infection, thus contributing to virus resistance [1,2]. However, another form of recruitment that occurs is the ability of plants to adapt host proteins with roles in normal growth and development to serve novel roles in resistance to plant pathogens. One example of this is the use of kinase signaling pathways that serve in growth, development, or ameliorating abiotic stress in mediating resistance responses [3]. Another example is the generation of novel plant effector molecules, which can either act directly on pathogens or affect their gene expression or translation products. Better-known plant effectors that are specifically associated with defense systems include proteins such as pathogenesis-related (PR) proteins [4], particular translation factors [5], and various components of the RNA silencing system, viz., Dicer-like endonucleases, Argonaute nucleases (“slicers”), and RNA-dependent RNA polymerases (RDRs) [6,7]. Lesser-known plant effectors include those induced during infection under specific circumstances, such as ribosome-inactivating proteins or various proteinaceous inhibitors of virus infection, initially by tobacco mosaic virus (TMV) in tobacco (*Nicotiana tabacum*) but often expanded to other viruses (rev. in [8]).

Among those proteins induced by TMV infection that were shown to have an antiviral role, the antiviral factor (AVF) and the inhibitor of virus replication (IVR) are the best-characterized. AVF was described by Sela and Appelbaum [9,10] as a family of phosphorylated glycoproteins stimulated by TMV infection in tobacco, one of which, gp35, is a β-1,3-glucanase, whereas another, gp22, is an isoform of the PR-5 protein (a thaumatin-like protein, also known as PR-S in earlier papers [4]) [11,12]; however, PR-S was shown not to have any antiviral activity when overexpressed in transgenic tobacco plants [13], whereas AVF was shown to inhibit TMV infection of tobacco if previously mixed with virus [9]. AVF was reported to function by stimulating plants to produce nucleotides with antiviral activities [14]. The regulation of the expression of the tobacco *AVF* gene has not been examined.

IVR was first described by Loebenstein and Gera [15], who infected tobacco protoplasts made from the TMV-resistant cultivar Samsun NN (SNN tobacco hereafter) with TMV. Then, they showed that culture fluid contained a substance that could inhibit TMV infection of protoplasts from either SNN tobacco or the susceptible tobacco without the *N* gene, cultivar Snn. The substance was not produced from either healthy SNN tobacco protoplasts or healthy or TMV-infected Snn tobacco protoplasts and was therefore specifically produced by TMV infection in protoplasts containing the *N* gene. The substance could be applied up to 18 h post inoculation (hpi) with TMV, showing that it inhibited replication of the virus and not uptake. In addition, IVR could be concentrated and fractionated by gel filtration into two active peaks of 26 kDa and 57 kDa, suggesting that it may form dimers [15]. Subsequent work with IVR showed that it could be recovered from protoplasts directly; could inhibit virus synthesis in leaf disks, as well as on sprayed leaves; could inhibit infection by cucumber mosaic virus (CMV) (in SNN tobacco and cucumber) and potato virus X (in *N. glutinosa*); and could not be induced by CMV in SNN tobacco [16]. In addition, IVR was not able to affect TMV directly and was insensitive to treatment with RNase but was sensitive to treatment with proteases and heating (60°C/10 min) and was therefore probably proteinaceous [16]. The TMV-induced synthesis of IVR in tobacco NN protoplasts was sensitive to treatment with the antimetabolites actinomycin D and chloramphenicol applied at either 5 or 24 hpi but insensitive to the same treatment applied at 48 hpi [17], indicating that IVR required RNA and protein synthesis for its production. The discovery that IVR could be collected from the intercellular fluid of non-inoculated parts of TMV-inoculated leaves, where TMV was applied either in parallel trips or at the base of the leaves, and could be collected (albeit in lesser quantities) from systemically-infected leaves made the procurement of larger amounts of enriched IVR easier [18]. Antisera prepared with the two IVR fractions (isolated from protoplast culture medium) reacted identically, suggesting that the 57 kDa protein was a dimer of the 26 kDa protein [18,19]. Antisera against IVR could neutralize the inhibitory activity of IVR but did not react with either AVF or human interferon, indicating that these all were different antiviral factors [19]. Polyacrylamide gel electrophoresis fractionation of the precipitated culture medium from TMV-infected SNN tobacco protoplasts produced a 23 kDa protein that exhibited IVR activity, which also could be neutralized by antiserum prepared against this protein [20].

Reciprocal interspecific crosses made between *N. glutinosa* and *N. debneyi* produced amphidiploid hybrids that showed strong resistance to infection by TMV, producing much smaller local lesions than those produced on *N. glutinosa* (~93% reduction in size), from which little if any infectious virus could be recovered [21]. These plants also constitutively produced a PR protein designated as b_1″_ [21,22], which was produced in both parent plants only after virus infection (TMV in *N. glutinosa* and tobacco necrosis virus in *N. debneyi*). The b_1″_ protein later was considered a member of the PR-1 class of PR proteins [23]. Thus, these plants appeared to be primed for expression of proteins associated with resistance. In addition, the resistance to TMV was not abrogated by incubation at 30 °C but required 35 °C for the loss of resistance [22]. When hybrid plants of the *N. glutinosa* × *N. debneyi* cross (designated H9) were examined for IVR expression with and without TMV infection, in both cases, IVR was detected by serological assays, showing that the IVR present in H9 plants was indistinguishable from the IVR in SNN tobacco [24]. Extracts from H9 leaves were assayed in SNN tobacco leaf disks and protoplasts and were shown to be inhibitory to infection by TMV using a local lesion assay, demonstrating the presence of functional IVR in the H9 plants [24]. When the H9 plants were incubated at 35 °C, TMV was able to infect those plants systemically, as it did in SNN tobacco plants, whereas IVR could not be detected in either set of plants maintained at 35 °C [25]. Thus, IVR expression is also regulated by the temperature-sensitive expression of other defense genes in *N*-gene *Nicotiana* species [26,27].

A cDNA clone of IVR from SNN tobacco was selected by phagemid cloning and screening for the expressed IVR protein with antisera to the 23 kDa protein. The cDNA clone (designated NC330) was 1016 bp and contained an open reading frame (ORF) encoding a 199-amino-acid (aa) protein, with a mass of 21,651 Da [28]. A probe generated from the cDNA clone showed that RNA of the same size was detected in TMV-infected SNN tobacco but not in non-inoculated SNN tobacco leaves, in mock-inoculated Snn tobacco or in TMV-infected Snn tobacco. The *E.coli*-expressed protein interacted with the anti-23 kDa serum but migrated farther than the IVR obtained from TMV-infected Samsun NN protoplasts, with an apparent mass of ~21 kDa. Nevertheless, the expressed IVR-like protein also showed the ability to inhibit virus replication in TMV-infected Snn tobacco leaf disks [28]. Transgenic expression of the IVR cDNA clone in Snn tobacco behind a constitutive cauliflower mosaic virus 35S promoter led to some plants in each of four generations examined showing expression of the IVR-like protein and resistance to infection by TMV, although homozygous, resistant, IVR-expressing transgenic plants apparently could not be obtained [29]. Plants showing the presence of IVR mRNA were not necessarily resistant, and highly resistant plants could produce plants that segregated for resistance and susceptibility, even hypersusceptibility (producing more TMV than the controls). In addition, IVR-expressing transgenic plants became susceptible to TMV infection when grown above 30 °C, and IVR transgenic seeds germinated poorly or not at all in the dark or at 34 °C [29], indicating unusual early developmental effects due to constitutive expression of IVR.

Infection of SNN tobacco by TMV or potato virus Y (PVY) induced the expression of several other genes involved in defense responses, including a mitochondrial alternative oxidase, the transcriptional factor (TF) signaling hub effector 1 (SHE1, previously known as ERF5 [30]), the RNA silencing signal amplifying enzyme RDR6, and IVR [31], as well as TF MYB1 [27,32] and peroxidases [32]. The transgenic silencing of the phytohormone-induced *RDR1* gene in SNN tobacco showed inhibitory effects on the PVY-induced expression of those defense response genes. The silencing of these genes resulted in an increase in the accumulation of PVY [31,32]. SHE1 was shown to be involved in resistance to TMV, with transgenic overexpression of SHE1 enhancing resistance in SNN tobacco; however, inducible SHE1 was not activated at 32 °C [30]. Recently, we found that IVR interacted with SHE1 and that silencing of SHE1 in SNN tobacco inhibited the TMV-induced expression of IVR, whereas constitutive overexpression of SHE1 led to constitutive expression of IVR [33], suggesting that SHE1 is a TF in the pathway producing IVR. Here, we further characterize IVR and consider its origin from a plant factor involved in the regulation of mitosis.

## 2. Materials and Methods

### 2.1. Yeast Two-Hybrid Assays

The Gateway System (Invitrogen, Carlsbad, CA, USA) was used to introduce RT-PCR products into the expression clones via the Gateway LR reaction (Invitrogen) using pAS-attR(BD) and pACT2-attR(AD), as described previously [34]. The primers used to generate the RT-PCR products are listed in Table S1. Yeast cells were cotransformed and interactive proteins growing in yeast cells were selected as described previously [34], using the YEASTMAKER Yeast Transformation System 2 Kit and User Manual (PT1172-1, Clontech, Mountain View, CA, USA). Transformants were selected on restrictive media plates without X-gal, lacking various amino acids or adenine, to detect transcription of reporter genes for HIS, LEU, TRP, ADE, and MEL1 [34].

### 2.2. Comparisons of DNA and Protein Sequences

The NCBI BLAST (https://blast.ncbi.nlm.nih.gov/Blast.cgi) and Dbfetch (http://www.ebi.ac.uk/Tools/dbfetch/dbfetch/) systems was used to obtain sequences of *IVR*-like genes and anaphase-promoting complex 7 (*APC7*)-like genes, as well as sequences of their encoded proteins, and perform alignments of sequences. Before analysis, the tobacco IVR sequence was edited to correct errors causing a frame shift between nucleotides 491 and 558, corresponding to the C-terminal region amino acids 164–175 and 186 [33].

### 2.3. Protein Structure Modeling by AlphaFold

IVR and APC7 protein were modeled using the AlphaFold program [35] with a user-friendly interface for accessing AlphaFold2 provided online via Github notebooks (https://colab.research.google.com/github/deepmind/alphafold/blob/main/notebooks/AlphaFold.ipynb#scrollTo=XUo6foMQxwS2, accessed on 8 May 2022), the structural prediction of which is supported by AlphaFold2 combined with a fast multiple sequence alignment generation stage using MMseq2 [36,37]. The modeling of homo- and heterocomplexes also was achieved using ColabFold.

The atomic coordinates and per-residue confidence estimates for predicted structures scale from 0 to 100. Higher scores are related to higher confidence. This confidence criterion is termed pLDDT (predicted local distance difference test) [38]. The pLDDT is defined in four levels: the first level includes high model confidence of residues with pLDDT ≥ 90; the second level shows the confidence model representing residues with 90 > pLDDT ≥ 70; the third level, representing residues with 70 > pLDDT ≥ 50, has low confidence; and the final level residues with pLDDT < 50 correspond to very low confidence [39]. The pLDDT is represented by a scale from red (bad) to blue (good) in AlphFold2 plots.

UniProt Blasts (https://www.uniprot.org/uniprotkb/, accessed on 28 July 2022) were performed with the IVR protein sequence; some of these that had 100% similarity with UniProt were used for AlphaFold predictions, which were performed with the APC7 from *Nicotiana sylvestris* (LOC104246274-anaphase-promoting complex subunit 7 isoform X2-Nicotiana sylvestris (Wood tobacco)|UniProtKB|UniProt, https://www.uniprot.org/uniprotkb/A0A1U7YEH5/entry, accessed on 28 July 2022).

### 2.4. Identifying TPR Elements

Tetratricopeptide repeat (TPR) elements present in IVR-like proteins and TPC7 proteins were identified from information provided in BLAST searches, as well as from data provided by the AlphaFold program [35] and as described in [40,41,42].

## 3. Results

### 3.1. IVR1 and MYB1 Interact with the CMV 1a Protein

**Figure 1:**
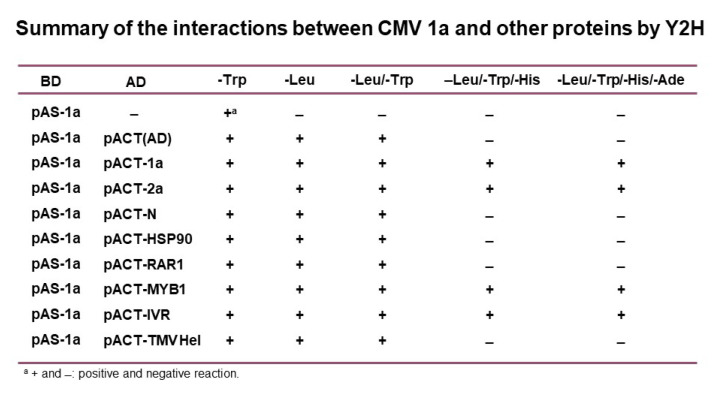
Yeast two-hybrid assay for interactions between the cucumber mosaic virus (CMV) 1a protein and various host defense factor proteins. The CMV 1a protein bound to the binding domain (BD) in the plasmid pAS was propagated in yeast cells, either alone or together with plasmids (pACT), expressing the activation domain (AD) fused to the CMV 1a protein, the CMV 2a protein (both positive controls), the TMV helicase domain of the 126K protein (negative control), the resistance gene protein N from tobacco, the defense chaperone proteins HSP90 and RAR1, the transcription factor MYB1, and the defense factor IVR. The metabolites listed at the top represent amino acids (Trp, Leu, and His) or the base adenine (Ade), which were missing for the plating media in which the yeast cells were grown. The pAS plasmid constitutively expresses Trp, and the pACT plasmid constitutively expresses Leu. Cells containing interacting test proteins induce the expression of both His and Ade. The growth of yeast cells on media-specific plates, indicating an interaction of the test proteins, is shown by “+”, whereas the absence of growth is indicated by “−“.

Previously, we showed that the CMV 1a protein could interact with the TF SHE1 in several systems, including the yeast two-hybrid system (Y2H) [34]. Here, we used the Y2H system to determine whether other early components of the *N*-gene-mediated defense response also interacted with the CMV 1a protein (Figure 1). Interactions between the CMV 1a replication-associated protein plus the CMV 2a replicase protein were used as positive controls, and the absence of an interaction of CMV 1a with the TMV helicase domain [43], which binds to the N protein, was used as a negative control for interaction with CMV 1a. CMV 1a also interacts with itself to form dimers [44]. Of the examined tobacco proteins, the N protein and the two chaperones (RAR1 and HSP90) that interact with each other and with the N protein via HSP90 [45] did not react with the CMV 1a protein, whereas IVR and the TF MYB1 did interact with the CMV 1a protein (Figure 1).

MYB1 is a TF that is activated by salicylic acid (SA) and TMV infection of *N*-gene tobacco [27] and is required for the *N*-gene-mediated resistance response to infection against TMV [46]. MYB1 binds to the PR1a promoter region but is a minor factor in the expression of the tobacco *PR-1a* gene, where TFs WRKY12 and TGA1a are the major regulators of PR-1a expression [47]. The consequences of the interactions of the CMV 1a protein with IVR and MYB1 is unknown but could be either part of the defense response or a counter-defense action by the CMV 1a protein. Here, we focused on the nature of the *IVR* gene and its encoded protein.

### 3.2. Sequence Analysis of IVR-like Proteins from Solanaceous Plants

**Figure 2:**
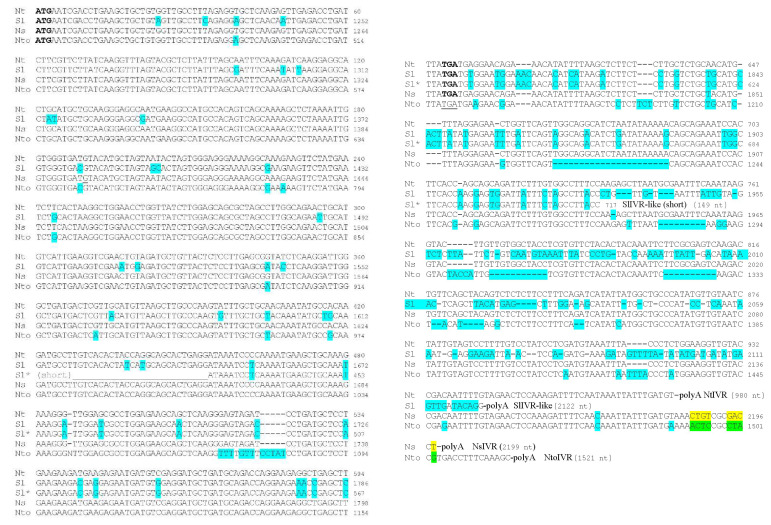
Nucleotide sequences of the *IVR*-like genes and 3′ nontranslated regions (3′ NTRs) of various cDNAs, expressed sequence tags, or mRNAs deduced from genomic DNA clones from the plant species tobacco (Nt) and tomato (Sl), as well as the two parent species of Nt, Ns, and Nto. Numbers to the right and at the end of the sequences refer to the positions of the sequence in Nt beginning at the start of the IVR open reading frame and, in the other species, the locations of the beginning of either cloned sequence or the beginning of the *IVR*-like gene within that cloned sequence. Differences between the Nt sequence and the other sequences are indicated by turquoise highlight. Differences between Ns and Nto beyond the 3′ NTR of the Nt sequence are indicated by yellow and green colors. The starting AUG and the terminating UGA are highlighted, except in Nto (where it is underlined), because the Nto mRNA sequence contains an insert, leading to a frameshift. “poly A” refers to the polyadenylated tail that follows the end of the 3′ NTR. Dashes were added to maintain the alignment when some sequences contained insertions relative to others.

At the time the *IVR* gene was sequenced [28] or expressed in transgenic tobacco SNN plants [29], none of the solanaceous genomes had been completely sequenced. Therefore, there were limited data available (1999–2005) for comparison of IVR sequences. An expressed sequence tag (EST) of 479 bp (GenBank Accession AW932904; 21 May 2001) obtained from tomato (*Solanum lycopersicum* cv. TA496) fruit as part of an unpublished study entitled “Generation of ESTs from tomato fruit tissue” was very similar (in antisense orientation) to part of the tobacco *IVR* gene from ORF nucleotide 454 to the termination codon; the 3′ nontranslated regions (NTRs) showed more differences, including insertions (Figure 2). Subsequently, a reference library of tomato cv. Micro-Tom [48] produced a tomato fruit cDNA with sequences very similar the tobacco IVR, although the sequence of the ORF was more than twice as long (GenBank Accession AK328373; 3 May 2010). The 3′ NTR sequences were identical to those of the shorter tomato EST shown in GenBank Accession AW932904 (Figure 2), but after the end of the shorter tomato EST, the longer tomato cDNA differed considerably in sequence from the tobacco IVR sequence to such an extent that it was no longer detectable by alignment tools (Figure 2).

Primers for PCR amplification of the tobacco IVR were used for RT-PCR on RNAs isolated from potato (*Solanum tuberosum*) cv. Phureja, which was cloned. The sequence of the ORF in the cloned PCR product was only 4 nt different from a 600 bp sequence within the genomic scf00035_44 of the potato cv. DM 1-3 516 R44 (AEWC01007128.1; 24 May 2011) and was very similar to the sequences of both the tobacco IVR (AJ009684) and the tomato Micro-Tom sequence (AK328373) (data not shown), as was the sequence of the encoded proteins (see below). *N. benthamiana* (*Nb*) also has sequences very similar the tobacco *IVR* gene (GIUP01022078.1; 4 October 2021). IVR-like sequences also were available from one of the parents of tobacco, *N. sylvestris* (*Ns*) (two identical, partial EST clones: BP744385 and BP745492; both 28 May 2004), which contained sequences of the IVR ORF from nucleotide 190 to the termination codon plus 167 sequences of the 3′ NTR. In this case, the limited 3′ NTR sequences between the *IVR*-like gene of *Ns* and *N. tabacum* (*Nt*) were nearly identical, containing one substitution, as well as three nucleotide deletions and five nucleotide insertions in *Ns* (data not shown). Because the *Ns* sequences were incomplete, the same analysis was performed again using mRNA sequences derived from the genomic DNA sequences of the *NsIVR*-like gene (XM_009802059.1; 21 October 2014) (Figure 2). In this case, there was only one deletion and one substitution in the 3′ NTR of *Ns*IVR-like RNA vs. the 3′ NTR of the *Nt*IVR RNA. In contrast, the sequences of the 3′ NTR of *Nt*IVR compared with the IVR-like sequences from the other tobacco parent, *N. tomentosiformis* (*Nto*) (XM_018778567; 20 April 2020), which also was derived from a genomic DNA clone, showed 22 substitutions, 12 insertions, and 60 deletions (52 in 4 blocks of 9-22 nt) in the *Nto*IVR-like 3′ NTR (Figure 2). Within the ORF of the IVRs, *Ns* showed no sequence differences from *Nt*, whereas *Nto* showed 15 substitutions and 6 inserts (Figure 2). Overall, the data indicate that the *IVR* gene of *Nt* tobacco is derived from the *Ns* parent.

**Figure 3:**
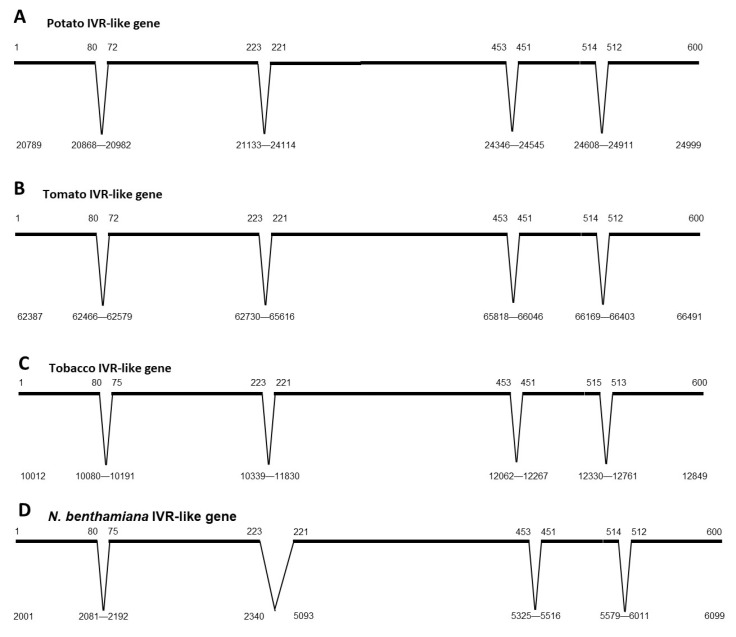
Genome organization of *IVR*-like genes in four solanaceous plants: (**A**) potato, (**B**) tomato, (**C**) tobacco, and (**D**) *N. benthamiana*. Based on genomic sequences that contain *IVR*-like genes and mRNA-derived sequences that contain IVR-like sequences, the positions of exons and introns could be determined. In many cases, additional copies were found, some containing minor base changes and others containing deletions or insertions, suggesting that dysfunctional copies are present in these genomes. This was particularly the case for *N. benthamiana* (see Figure S1). Numbers above the “chromosome line” represent the transcripts coordinates, and numbers below the lines or introns represent the genome sequence coordinates from specific genomic scaffolds.

The genomic sequences of *IVR*-like genes from *Nt*, tomato, potato, and *Nb* were compared and found to share many common features (Figure 3). The ORFs of the potato and tomato *IVR*-like genes (Figure 3A,B) were split into five exons in which the sizes of corresponding exons between species were almost identical. This was also the case for the four introns, among which the second intron was the largest. The *NtIVR* gene contained a similar arrangement of introns and exons, but the second intron was much smaller (1495 bp for tobacco vs. 2981 bp for potato and 2886 bp for tomato (Figure 3C vs. Figure 3A,B). In contrast, the *IVR*-like genes varied in size, with *Nt* having the shortest length (2837 bp), whereas potato (4210 bp) and tomato (4104 bp) were longer. In the case of *Nb,* the numbers and lengths of the exons were similar to those in the *NtIVR* gene (Figure 3C vs. Figure 3D), but the entire *Nb* gene was much longer and contained duplications of exons (Figure S1). Specifically, exons 1–3 were duplicated, separated by introns some 4000 bp further along the same BAC fragment after the end of the complete copy of the *IVR*-like gene (Figure S1B), although the second intron of the partially duplicated *IVR*-like gene was much shorter (703 bp). In addition, in the complete copy of the *IVR*-like gene, the second intron (2350 bp) contained an inverted copy of the last exon (Figure S1A), whereas upstream of the complete copy, there were other exons (or a fragment) inserted in inverted orientations (Figure S1C). It is not known whether the *IVR* gene is expressed in *Nb* during defense responses.

### 3.3. Sequence Analysis of IVR-like Proteins from Various Plant Families

**Figure 4:**
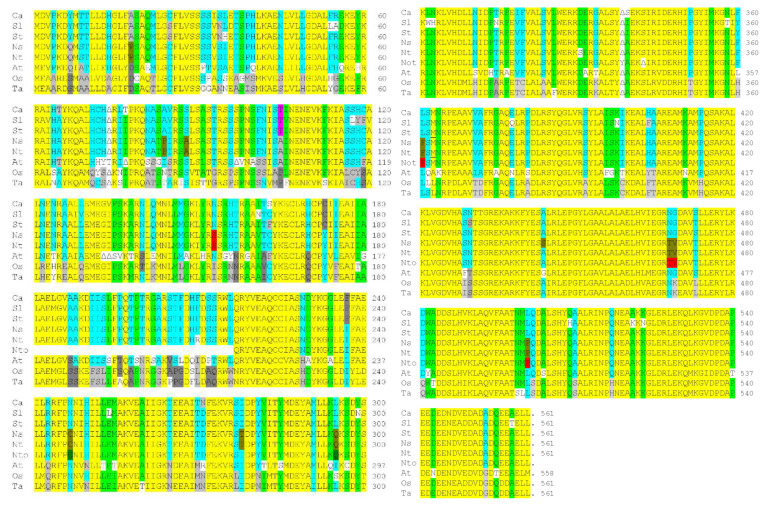
Amino acid sequence comparisons of anaphase-promoting complex (APC7) proteins from six solanaceous species, Arabidopsis, and two monocot grass species. The species examined were pepper (Ca), tomato (Sl), potato (St), *N. sylvestris* (Ns), *N. tabacum* (Nt), *N. tomentosiformis* (Nto), Arabidopsis (At), rice (Os), and wheat (Ta). Sequences were aligned with triangles inserted to maintain the alignments, where one or more amino acids were absent relative to the same position in other species. Similar sequences were grouped together; the similarity was based on type of amino acid: basic (H, K, and R), acidic (D and E), amide (N and Q), hydroxylated aliphatic (S and T), small aliphatic (A, L, I, M, and V), or phenyl group (F and Y). Not within groups are C, G, P, and W, as they all affect the folding of the protein. Highlight key: yellow—all sequences within a column were similar; light green—all but one of the amino acids in a column were similar; turquoise—the majority or a plurality of the amino acids in a column were similar; light or dark grey—two to four sequences in a column were similar; red—similar sequences found only in the *Nicotiana* species; and white—a novel amino acid at this position. Note: the sequence of the *Nto*APC7 appears to be lacking either the N-terminal 216 aa with no start codon (XP_009629102.1, derived from mRNA XM_009630807.1; 15 October 2014) or the N-terminal 254 aa beginning with the amino acid sequence “MEKVEA” (XP_018634083.1, derived from mRNA XM_018778567.2; 20 April 2020).

An *Arabidopsis thaliana* protein-coding sequence (At2g39090; 2 February 2001) encoding a 276 aa partial protein was identified, showing high sequence similarity to the 199 aa tobacco IVR sequence. A later sequence analysis identified this Arabidopsis coding sequence in a 558 aa protein (NP_850309; 16 September 2003) (Figure S2) containing TPRs, which are involved in protein–protein interactions [44], and showing sequence similarity to both human and mouse anaphase-promoting complex subunit 7 (APC7). A comparison of the corrected tobacco IVR protein (from cv. Samsun NN) sequence against the *Nt*APC7 protein sequence (XP_016480892.1; 3 May 2016) (from cv. TN90) showed that in their overlap regions, they differed by only one amino acid (APC7 amino acid 444 is alanine, whereas the corresponding *Nt*IVR amino acid (82) is serine), indicating that IVR is derived from APC7. A comparison of *Nt*APC7 against those of the APC7 of the parental species from which *Nt* is derived (*Ns* and *Nto*) showed that there were minor differences in sequence between all three proteins (Figure 4), with few differences in sequence between *Nt*APC7 and *Ns*APC7 (positions 150, 210, 331, and 444) and between *Nt*APC7 and *Nto*APC7 (positions 278, 339, and 342/343).

A further comparison of the *Nicotiana* spp. APC7 sequences with other solanaceous species, namely tomato, potato, and pepper (*Capsicum annuum*), as well as with *At*APC7 and the sequences of APC7 from two monocotyledonous species, rice (*Oryza sativa*) and wheat (*Triticum aestivum*) (Figure 4), showed a remarkable degree of conservation of sequence. A total of 67% of the amino acid sequences at any given position were similar (yellow highlight) among the nine plant species, with 12% of the sequences at any given position among the nine plant species containing only one non-conserved amino acid (light green) and 20% of the amino acids located at specific positions among the nine plant species similar either in a majority or plurality of occasions (turquoise). The methionine corresponding to the N terminus of the IVR is located at amino acid position 363 of most of the sequences; however, neither Arabidopsis nor the two monocot species contains a methionine at this position (Figure 4). In addition, either all three *Nicotiana* species or only *Ns* and *Nt* contained novel amino acid selections at 12 positions (red), whereas the amino acid sequences at 59 positions were unique to the two monocot species (Figure 4).

### 3.4. Analysis of the TPR Units in Tobacco IVRs vs. At*APC7*

**Figure 5:**
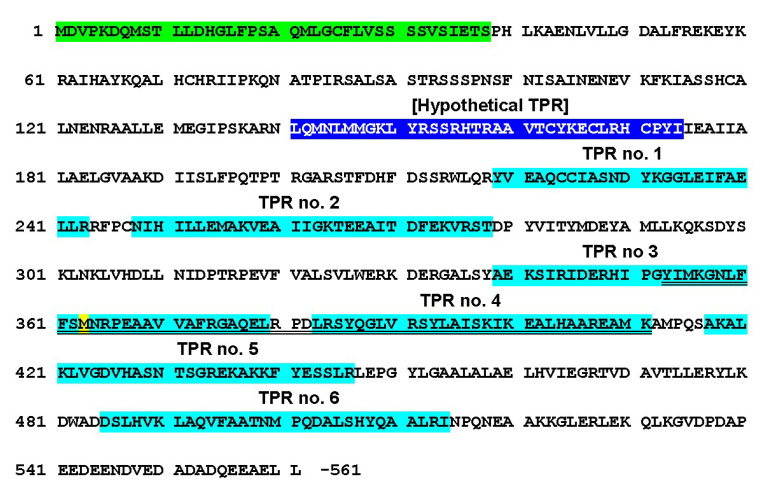
Mapping of tetratricopeptide repeat (TPR) units and dimerization domain of anaphase-promoting complex (APC7) on the amino acid sequences of ACP7 from *Nicotiana sylvestris* (*Ns*). Highlight key: light green—dimerization domain predicted from human APC7 [42]; dark blue—hypothetical TPR before TRP no. 1; turquoise—predicted *Ns*TPR unit no. 1 (amino acids 219–243), no. 2 (amino acids 248–278), no. 3, (amino acids 339–379), no. 4 (amino acids 383–411), no. 5 (amino acids 417–446), and no. 6 (amino acids 485–514); and yellow—methionine start codon for IVR. The double-underlined sequences represent a double TPR unit designated TPR11.

TPR units contain ~34 aa and form a helix–turn–helix structure [41]. The structure of *At*APC7 was predicted to contain 10 TPR units. Of these, the *Ns*APC7 was predicted to contain six TPR units (Table 1 and Figure 5; XP_009800361.1; 21 October 2014). However, the human APC7 was shown to contain noncanonical TPR units, in which some of the conserved sequences were not always present at the designated locations in the TPR units [42]; in particular, in the TPR motif, x_3_Wx_2_LGx_2_Yx_8_Ax_3_Fx_2_Ax_4_P, W-4 was always absent, and L-7 and G-8 were usually absent. It is difficult to rationalize why some of the TPR units are thus identified, given that many did not have most of the canonical motif sequences present. This applies to TPR units identified in both tobacco and Arabidopsis, as well as such sequences identified as TPR units in Arabidopsis but not identified as such in the tobacco species. Specifically, the first *At*APC7 TPR unit (Table 1) had only one conserved amino acid of this motif, which was also the case for the similar *Nicotiana* APC7 sequences (Figure 4 and Figure 5). In the case of the second *At*APC7 TPR unit (Table 1), the corresponding sequences of the three *Nicotiana* APC7 proteins contained five of the eight canonical TPR motif sequences, whereas the *At*APC7 sequence contained only three of the eight canonical TPR motif sequences (Figure 4 and Figure 5). In the third *At*APC7 TPR unit, equivalent to the first *Ns*APC7 unit, the *At*PC7 corresponding sequences only contained three of the eight canonical TPR motif sequences, whereas the three *Nicotiana* APC7 proteins only contained two of the eight canonical TPR motif sequences (Figure 4 and Figure 5). The fourth *At*APC7 TPR unit and the equivalent second *Nicotiana* APC7 units, as well as the fifth *At*APC7 TPR unit, all contained only three of the eight canonical TPR motif sequences. The third *Nicotiana* APC7 TPR units, which overlapped with part of the fifth and all of the sixth *At*APC7 TPR units, contained six of the eight canonical TPR motif sequences, whereas the sixth *At*APC7 TPR unit only contained four of the eight canonical TPR motif sequences, including the terminal proline. This is also the motif in which the *Nicotiana* IVR sequences began (Figure 4 and Figure 5). The seventh *At*APC7 TPR unit and the equivalent fourth *Nicotiana* APC7 TPR units both contained six of the eight canonical TPR motif sequences. The eighth *At*APC7 TPR unit and the equivalent fifth *Nicotiana* APC7 TPR unit both contained only three of the eight canonical TPR motif sequences (Figure 4 and Figure 5). The ninth *At*APC7 TPR unit with no *Nicotiana* equivalent contained no prolines in this region or adjacent to this region and only had one or two of the other TPR sequences aligned (Figure 4 and Figure 5). Finally, the tenth AtAPC7 TPR unit and the equivalent sixth *Nicotiana* APC7 TPR unit had five and six of the eight canonical TPR motif sequences, respectively (Figure 4 and Figure 5).

### 3.5. Structural Analysis of the Nt*IVR*

**Figure 6:**
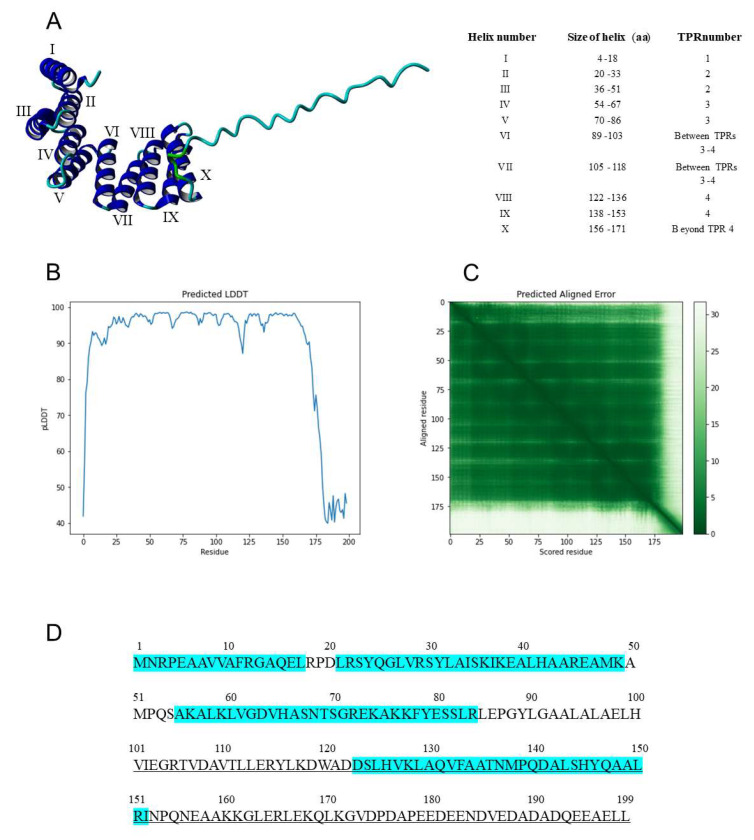
Three-dimensional structure and protein sequence of IVR. (**A**) (Left) 3D structure for IVR modeled by AlphaFold, showing 10 helices (from the N terminus, identified by Roman numerals) followed by a disordered random structure for the C-terminal 28 aa. The C terminus is at the far right of the molecule. (Right) Table showing the locations of the 10 helices in the IVR sequence, as well as their positions with regard to the IVR TPRs. (**B**) The predicted local distance difference test (pLDDT) and (**C**) the predicted aligned error (PAE) scores are shown. The pLDDT is a per-residue confidence metric (scale of 1–100). Based on the pLDDT and PAE indices, the predicted 3D structure derived from the protein sequence of IVR has high confidence scores for the domains at residues 1-174 (green in C) but not with the region between 175 and 199 (the C terminus; white in C). The predicted IVR structure in which IVR amino acids 1 and 2 (M and R), 19(P), 33 (I) to 34 (S), 52 (P) to 53 (Q), 67 (S) to 70 (S), 87 (P) to 88 (G), 119 (D) to 121 (A), 154 (Pro) to 155 (Q), and 172 (G) to 199 (L) are confident (pLDDT 70-90), but other amino acids show very high pLDDT indices (pLDDT > 90). (**D**) Amino acid sequence of IVR, with TPR units highlighted in turquoise. The methionine initiation codon is at position 1. Sequences within underlined C-terminal 99 aa were found to contain a binding domain to the SHE1 transcription factor [33].

The AlphaFold program [35] was used to model the three-dimensional (3D) structure of IVR. The information explaining the levels of confidence in this structure are given in Section 2.3 and shown in Figure 6B,C. This structure contains 10 helical regions (Figure 6A) from amino acid 5 to amino acid 171 (bordered by prolines and at the edges of TPR units), including two α helices (nos. VI and VII) between TPR units 3 and 4 and another α helix (no. X) after TPR unit 4, followed by a C-terminal 28-amino-acid random structure (Figure 6A,B). These helical regions include two each in three TPR units (nos. 2, 3, and 4 in Figure 6D, equivalent to TPR nos. 4, 5, and 6, respectively in Figure 5) and one in the part of the split APC7 TPR no. 3 (Figure 5) located in the N-terminal 17 aa of IVR (Figure 6D).

### 3.6. Structural Analysis of the Nt*IVR* vs. At*APC7*

**Figure 7:**
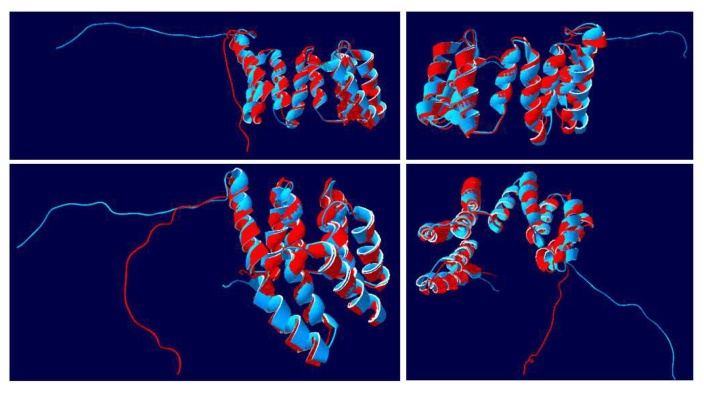
Superimposed 3D structures of NtIVR with the C-terminal 201 aa of Arabidopsis anaphase-promoting complex (*At*APC7). The structure of the 199 aa tobacco IVR sequence (blue) was modeled together with the structure of *At*APC7 amino acids 361-561 (red). Four views for each superimposition of the two structures are shown, displaying the alignment of various helical units, as well as positions of the C-terminal 28 aa random strands.

The 3D structure of *At*APC7 was modeled (AlphaFold DB Q8VY89), as was the structure of the *Ns*APC7 (LOC104246274-anaphase-promoting complex subunit 7 isoform X2-Nicotiana sylvestris (Wood tobacco)|UniProtKB|UniProt, https://www.uniprot.org/uniprotkb/A0A1U7YEH5/entry, accessed on 28 July 2022). The structure of the 199 aa *Nt*IVR (blue) of similar sequence was modeled together with the C-terminal 201 aa of *At*APC7, i.e., amino acids 361-561 (red) (Figure 7). In four different views of the superimposed structures, the two molecules were identical, except for the non-structured C-terminal 28 aa (Figure 7). Nine of the ten helices were readily discernable, but due to overlapping by the helical bundles, a tenth helix is difficult to discern in various views. The superimposition showed that there were no differences in the number, size, or position of helices, indicating that the 85-122 and 152-171 regions of IVR amino acids must also form helices. This supports the conclusion that IVR is derived from APC7.

**Figure 8:**
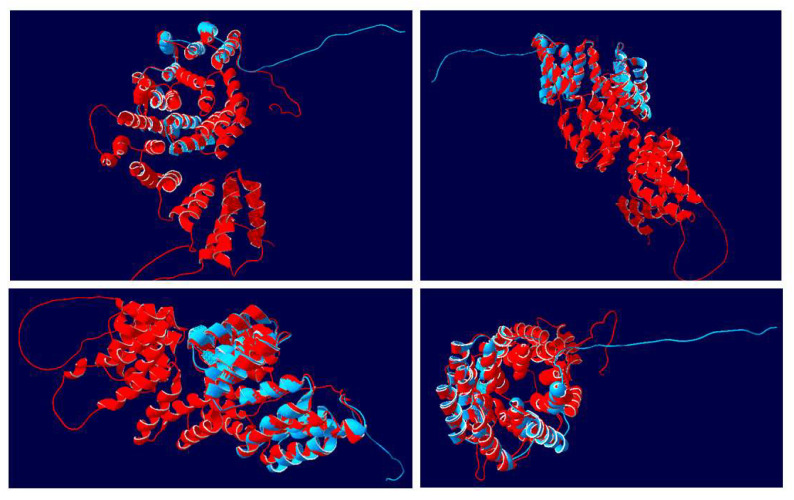
Superimposed 3D structures of NtIVR with that of the complete Arabidopsis anaphase-promoting complex (AtAPC7). The structure of the 199 aa tobacco IVR sequence (blue) was modeled together with the structure of the 561 aa AtAPC7 (red). Four views for each superimposition of the two structures are shown, displaying the alignment of various helical units (as shown in Figure 7) and the separate N-terminal 360 aa domain of AtAPC7 (red), as well as the positions of the C-terminal 28 aa random strands.

When the complete *At*APC7 sequence was superimposed on the *Nt*IVR sequence in the model (Figure 8), it was clear that the *At*IVR molecule largely formed two discrete domains. The N-terminal 360 aa formed a cluster of helical bundles generated from various TPR units plus other helices and large looped regions between several of the helical bundles, as described for human APC7 [42]; beyond this region of the molecule were helical bundles in the superimposed regions, corresponding to the alignment of structures between *At*APC7 and *Nt*IVR, as shown in Figure 7.

## 4. Discussion

The data presented here showed that IVR, a long-studied antiviral factor from tobacco [49], was able to interact with the CMV 1a protein, just as IVR interacted with the TF SHE1 [33] and the SHE1 and CMV 1a proteins interacted with each other [34]. To gain a better understanding of the nature of the IVR, we examined the sequences of *IVR*-like genes in a number of plant species and identified a larger protein, APC7, that contained the IVR-like sequences in the C-terminal 36% of the APC7 protein (Figure 4 and Figure 5). In addition, the protein sequences encoded by the *APC7* gene are highly conserved in many species ([42]; Figure 4). APC7 contains numerous TPR units, which are involved in interactions with other proteins, usually with the C-terminal region of those proteins, in one or more of several motifs, such as the isoleucine–arginine (IR) motif [42]. APC7 is a component of the cellular cyclosome (aka APC), a E3 ubiquitin ligase controlling the progression of mitotic division [46]. APC is composed of at least 13 subunits, including a cullin homolog (APC2), a ring-H2 finger domain (APC11), a Doc domain protein (APC10) with an IR motif that promotes substrate binding, and four subunits containing TPR elements (APC 3, 6, 7, and 8). APC also requires two adaptor proteins (Cdc20 and Cdh1, both containing WD40 domains). The adaptors and various subunits permit recognition of substrates [50]. APC4 and APC5 are required for the TPR subunits to bind to APC1 [46,47]. The C-terminal halves of APC7 and APC3 contain TPR units needed for the binding of the adaptor Cdc20 and the substrate Nek2A, which interact with the TPR units via their IR peptides [51]. In addition, CP7 subunits form dimers through the N-terminal 38 aa dimerization interface [42].

Modeling of the *Nt*IVR protein structure, both by itself (Figure 6) and superimposed on either the corresponding region of APC7 (Figure 7) or the complete APC7 molecule (Figure 8), showed that except for the C-terminal 28 aa in both proteins, the two molecules were of identical structure in their overlapping regions. IVR lacks the dimerization domain present near the N terminus of APC7 and, except in Arabidopsis, does not contain any cysteine residues in the IVR sequence (Figure 4). Thus, it seems likely that IVR could still form complexes with some of the other components of the APC, as well as with some adaptor and substrate proteins. Whether this would be sufficient to activate the ubiquitin ligase components of the APC is not known. If it could, IVR might have some role in proteasome targeting, albeit with a different specificity than for the APC. Such an altered specificity might be suitable for targeting specific viral encoded proteins.

IVR was considered to form a dimer based on two peaks of antiviral activity detected by gel filtration chromatography, with 74% inhibition activity associated with the monomer unit (ca. 26-27 kDa) and 37% with the dimer unit (56-57 kDa) [15]. This suggests that either the dimer unit was less active than the monomer unit or that most of the protein was in monomer form. We could not detect self-interaction of IVR in the Y2H system (data not shown). Thus, we suggest that the original dimer-sized activity detected by Loebenstein and Gera [15] may have been caused by the presence of the full-length 62.8 kDa APC7 rather than a dimer of the 21.6 kDa IVR monomer.

Because IVR has the same sequence and structure as the C-terminal 201 aa of APC7 (Figure 4, Figure 5, Figure 6, Figure 7 and Figure 8), it seems likely that IVR was generated via the *APC7* gene. The question remains as to how IVR is formed. Is the APC7 protein processed to form IVR by some specific protease cleavage, or is IVR mRNA transcribed from transcription start sites present upstream of the IVR coding sequence in the *APC7* gene? The third option—that IVR is the result of alternative splicing of the APC7 transcript—is unlikely, as there are no additional sequences at the 5’ NTR of the IVR mRNA that are derived from sequences further upstream in the *APC7* gene. Although we cannot rule out protein processing of APC7 to produce IVR, we note that IVR mRNA of ca. 1000 nt was detected by northern blot hybridization of gel-fractionated RNAs extracted from TMV-induced SNN tobacco but not RNAs of uninduced SNN tobacco [28]. This suggests that the most likely origin of IVR mRNA is by transcription from transcription starts sites upstream of the IVR coding sequence, at least in solanaceous plants.

**Figure 9:**
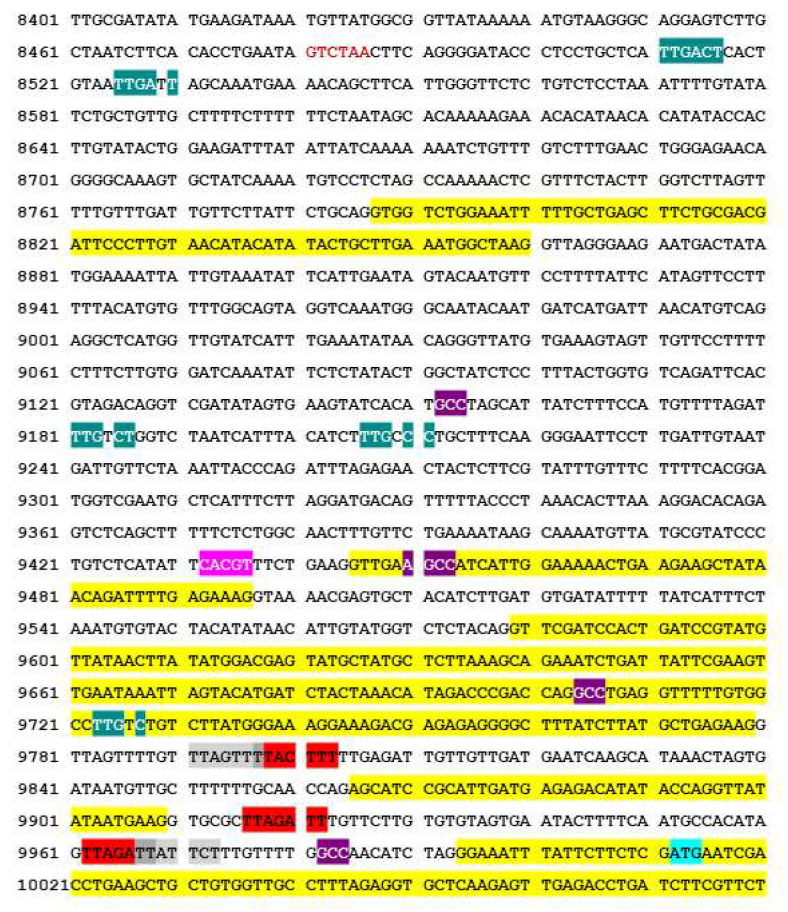
Putative transcription start sites and promoter sites for transcription of the *Nt*IVR mRNA within the *NtAPC7* gene (cv. K326) from accession AWOJ01567524 (28 April 2015). Yellow highlighted regions constitute exons from upstream APC7 sequences, with the bottom one shown containing the IVR initiation start codons ATG (in turquoise highlight); introns are not highlighted. Putative promoter sites: WRKY TF binding sites [TTGACC/T] (teal highlight); GCC element of ethylene-responsive element-binding protein (EREBP) TF binding sites [AGCCGCC] (violet highlight); and YACGTGG/TC-like ABA-responsive element-binding protein site (ABRE) sequence (blue). Putative transcription start-site sequences [YYAN(T/A)YY], where transcription starts with the underlined A (in red or light grey highlight, with dark grey highlight for shared nucleotides in overlapping start sites). Note that some promoters and transcription start sites are located within introns, which are less conserved between species.

Previously, we showed that IVR and the TF SHE1 were both induced during TMV infection in NahG tobacco plants, expressing the enzyme salicylate dehydroxylase [33], which inhibits the accumulation of SA [52]. In addition, silencing or overexpressing the *SHE1* gene caused a parallel change in the expression of IVR [33]. This led us to suggest that SHE1 was a TF involved in expression of IVR, either by binding to a promoter region upstream of the *IVR* gene or further upstream. SHE1 was found to bind weakly to the GCC sequence, an element of the ethylene-responsive element-binding protein (EREBP) site [30]. However, unlike other ethylene-responsive TFs, SHE1 (formerly *Nt*ERF5) was not induced by ethylene, SA, or jasmonic acid [30]. Therefore, we examined the sequences upstream of the IVR coding sequence in the *NtAPC7* gene for possible EREBP sites. Four such sites were identified: 28–30 bp, 306–308 bp, 558–560 bp, and 857–859 bp upstream of the initiation codon of IVR (Figure 9). We also examined this region of the *NtAPC7* gene of ca. 1500 bp for other TF binding sites. We found one binding site for the YACGTGG/TC-like ABA-responsive element-binding protein site (ABRE) 576-580 bp upstream of the IVR initiation codon and five binding sites for WRKY TFs 284-289 bp, 801–806 bp, 826–831 bp, 1482–1487 bp, and 1496–1501 bp upstream of the IVR initiation codon (Figure 9). As we stated at the end of Section 3.1, it is not unusual for two or more TFs to work together (synergistically or additively [47]). Therefore, it is possible that more than one TF may be involved in the expression of IVR from the upstream sequences within the *APC7* gene. Future experiments will examine whether SHE1 can bind to sequences within this region of the *APC7* gene and whether other TFs are also involved in the expression of IVR.

To allow for expression of IVR from APC7, transcription start sites need to be present upstream of the translation initiation site. In the sequence shown in Figure 9, there are five potential transcription start sites located between 39 bp and 221 bp upstream of the translation initiation codon (shown in red and grey highlights). The first two and last two overlap in the canonical initiation site sequence, YYAN(T/A)YY.

If our conclusions are correct that IVR was generated by the repurposing a functional domain of a larger protein to provide additional defense functions, then this may not be the only such situation. There are many genes that are active only at specific times in the cell cycle, and they may be available for other functions during other times that would not interfere with their primary function.

## Figures and Tables

**Table 1:** Comparison of the predicted tetratricopeptide repeats (TPR) in AtAPC7 and NsAPC7.

*At*APC7		*Ns*APC7	
TPR No.	Borders (aa No.)	TPR No.	Borders (aa No.)
1	43–76		
2	138–171		
3	212–245	1	219–243
4	246–279	2	248–278
5	314–346		
6	348–380	3	339–379
7	381–413	4	383–411
8	414–448	5	417–446
9	450–482		
10	483–515	6	485–514

## Data Availability

The data presented in this study are available in the article and Supplementary Materials, as well as in online databases.

## Supplementary Materials

The following supporting information can be downloaded at: https://www.mdpi.com/article/10.3390/v14122782/s1, Table S1: Oligonucleotide primers used in reverse transcription-polymerase chain reaction amplification for the yeast two-hybrid assay; Figure S1: Genome organization of IVR-like genes in *Nicotiana benthamiana*; Figure S2: Comparison of an unknown protein-encoding gene fragment of *Arabidopsis thaliana* with the sequence encoding the full-length Arabidopsis anaphase-promoting complex subunit 7 (APC7). References [53,54,55,56,57] are cited in the supplementary materials.
