# Supplementary material for: The Secret Life of the Inhibitor of Virus Replication
Source: Viruses. 2022 Dec 14;14(12):2782. doi: 10.3390/v14122782 (PMC9787567; doi:10.3390/v14122782)
Supplement: Supplementary file 1 [file viruses-14-02782-s001.zip › viruses-2066841-supplementary.pdf]

Supplementary Table and Figures

The Secret Life of the Inhibitor of Virus Replication

Peter Palukaitis [1,*], Masoud Akbarimotlagh [2], Eseul Baek [1], and Ju-Yeon Yoon [3,4,*]

1 Department of Horticulture Sciences, Seoul Women's University, Seoul 01797, Republic of Korea

2 Plant Pathology Department, Faculty of Agriculture, Tarbiat Modares University, Tehran 14115-111, Iran

3 Department of Plant Protection and Quarantine, Jeonbuk National University, Jeonju 54896, Republic of Korea

4 Department of Agricultural Convergence Technology, Jeonbuk National University, Jeonju 54896, Republic of Korea

* Correspondence: scripath1@yahoo.co.uk (P.P.); juyeon74@gmail.com (J.-Y.Y.); Tel.: +82-63-270-4188 (J.-Y.Y.)

Table S1. Oligonucleotide primers used in reverse-transcription-polymerase chain reaction amplification for the yeast two-hybrid assay

Name	Accession No.	Size (bp)	Primer sequence	Reference
CMV-1a	D00356	2982	F: 5'-gggg-acaagtttgtacaaaaaagcaggctcc**atggcgacgtcctcgttcaa**-3' [a,b]<br>R: 5'-gggg-accactttgtacaagaaagctgggt**ctaagcacgagcaacacatt**-3'	[53]
CMV-2a	D00355	2574	F: 5'-gggg-acaagtttgtacaaaaaagcaggctcc**atggctttccctgcccccgc**-3'<br>R: 5'-gggg-accactttgtacaagaaagctggg**tcagactcgggtaactccgc**-3'	[54]
N gene	U15605	3432	F: 5'-gggg-acaagtttgtacaaaaaagcaggct**ccatggcatcttcttcttcttc**-3'<br>R: 5'-gggg-accactttgtacaagaaagctggg**tcagcaaaggcgaagatgtt**-3'	[55]
HSP90	AY519499	2100	F: 5'-gggg-acaagtttgtacaaaaaagcaggctcc**atggcggatacagagacgtt**-3'<br>R: 5'-gggg-accactttgtacaagaaagctggg**ttagtcaacctcctccatct**-3'	None
RAR1	AF480487	666	F: 5'-gggg-acaagtttgtacaaaaaagcaggctcc**atggagagacttcgttgcca**-3'<br>R: 5'-gggg-accactttgtacaagaaagctggg**ttaggacgctgggctggcgt**-3'	[56]
MYB1	U72762	837	F: 5'-gggg-acaagtttgtacaaaaaagcaggctcc**atggtgagagctccttgttg**-3'<br>R: 5'-gggg-accactttgtacaagaaagctggg**tcaaaattctggtaattctg**-3'	[27]
IVR	AJ009684	600	F: 5'-gggg-acaagtttgtacaaaaaagcaggcttc**atgaatcgacctgaagctgc**-3'<br>R: 5'-gggg-accactttgtacaagaaagctggg**tcataaaagctcagcctctt**-3'	[28]
TMV-Hel	NC_001367	1341	F: 5'-gggg-acaagtttgtacaaaaaagcaggctcc**atggagatagagtctttaga**-3'<br>R: 5'-gggg-accactttgtacaagaaagctgggt**ctattgtgttcctgcatcga**-3'	[57]

[a] Underlined characters are start and stop codons.
[b] Bold characters are recombination sites for using the Gateway system.

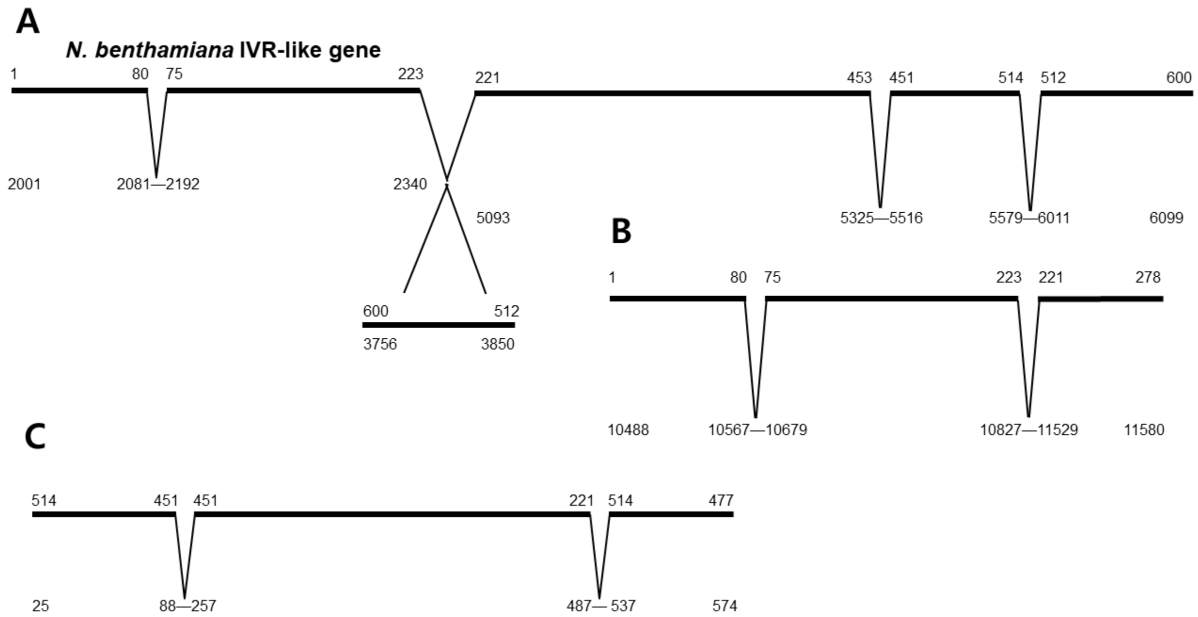

Figure S1. Genome organization of IVR-like genes in *Nicotiana benthamiana*. Genomic sequences and mRNA-derived sequences that contain IVR-like sequences were compared with the sequence of the tobacco IVR coding region, allowing the positions of exons and introns to be determined (A). In *N. benthamiana*, alternate copies of partial or complete exons were found, either downstream of the complete IVR copy (B), upstream of the complete copy (C), or within an intron of the complete copy (A); those exons or fragments in either the intron in (A) or upstream in (C), were in antisense orientation. Numbers above the "chromosome line" represent the transcripts coordinates, while numbers below the lines or introns represent the genome sequence coordinates from specific genomic scaffolds.

A

MEVPKEQIAT LIEHGLYDSA EMLGCFLVSS PTVSAETSPQ LKAENLILLG DALFHQREHR 60
RAIHTYKQAL HHYTRIPKQS SGISRSSLSL STRSSVNASS ISAINENEVR FKIASSHFAL 120
NETKAAIAEM ESVKTRSLEM NILMAKLHRN SGYNRGAIAF YKECLRQCPY VLEAVIGLAE 180
LGVSAKDIIS SFTQTSNRSA KVSLDQIDPT RWLQRYVEAQ CCVASHAYKG ALELFAELLQ 240
RFPNNVHLLT ETAKVEAIIG KNDEAIMRFE KVRSIDPYTL TS 282

B

MDEYAMLLQI KCDYSRLNKL VHDLLSVDHT RAEVFVALSV LWERKDARTA LSYAEKSIRV 60
MDEYAMLLQI KCDYSRLNKL VHDLLSVDHT RAEVFVALSV LWERKDARTA LSYAEKSIRV 342

DERHIPGYIM KGNLLLQAKR PEAAAIAFRA AQNLRSDLRS YQGLVHSYLA FGKTKEALYT 120
DERHIPGYIM KGNLLLQAKR PEAAAIAFRA AQNLRSDLRS YQGLVHSYLA FGKTKEALYT 402

AREAMNAMPQ SAKALKLVGD VHAFTSSGRE KAKKFYESGL RLEPGYLGAV LALAELHLME 180
AREAMNAMPQ SAKALKLVGD VHAFTSSGRE KAKKFYESGL RLEPGYLGAV LALAELHLME 462

GRNGDAVSLL ERYLKDYADD SLHVKLAQVF AATNMLQDSL SHFQAALRIN PQNEAAKKGL 240
GRNGDAVSLL ERYLKDYADD SLHVKLAQVF AATNMLQDSL SHFQAALRIN PQNEAAKKGL 522

DRLEKQMKGI DPDATDENDE NDVEDVDGDT EEAELM (276)
DRLEKQMKGI DPDATDENDE NDVEDVDGDT EEAELM (558)

Figure S2. Comparison of an unknown protein-encoding gene fragment of *Arabidopsis thaliana* with the sequence encoding the full-length Arabidopsis anaphase-promoting complex subunit 7 (APC7). (A) The N-terminal 282 amino acids (aa) sequence of the 558-aa APC7 protein (NP_850309; 16 SEP 2003), external to the C-terminal co-linear fragment alignment. (B) Comparison of the partial 276-aa APC7 protein sequence (derived from At2g39090; 02 FEB 2001) (upper line of each paired lines) with the C-terminal 276-aa APC7 protein sequence of the 558-aa APC7 protein (lower line of each paired lines). The two sequences are identical in the regions of overlap.
